# Genomic Characterization of a Uropathogenic *Escherichia coli* ST405 Isolate Harboring *bla*_CTX-M-15_-Encoding IncFIA-FIB Plasmid, *bla*_CTX-M-24_-Encoding IncI1 Plasmid, and Phage-Like Plasmid

**DOI:** 10.3389/fmicb.2022.845045

**Published:** 2022-04-11

**Authors:** Mianzhi Yao, Qianhui Zhu, Jin Zou, Abebe Mekuria Shenkutie, Songnian Hu, Jiuxin Qu, Zilong He, Polly H. M. Leung

**Affiliations:** ^1^Department of Health Technology and Informatics, The Hong Kong Polytechnic University, Hung Hom, Hong Kong SAR, China; ^2^State Key Laboratory of Microbial Resources, Institute of Microbiology, Chinese Academy of Sciences, Beijing, China; ^3^College of Life Sciences, University of Chinese Academy of Sciences, Beijing, China; ^4^Department of Clinical Laboratory, Shenzhen Third People’s Hospital, Second Hospital Affiliated to Southern University of Science and Technology, National Clinical Research Center for Infectious Diseases, Shenzhen, China; ^5^Department of Microbiology, Immunology, and Parasitology, St. Paul’s Hospital Millennium Medical College, Addis Ababa, Ethiopia; ^6^Beijing Advanced Innovation Center for Big Data-Based Precision Medicine, Interdisciplinary Innovation Institute of Medicine and Engineering, Beihang University, Beijing, China; ^7^School of Engineering Medicine, Beihang University, Beijing, China

**Keywords:** uropathogenic *Escherichia coli*, comparative genomics, plasmids, *bla*_CTX-M_, mobile genetic elements

## Abstract

*Escherichia coli* sequence type 405 is an emerging antibiotic-resistant clonal group associated with the global dissemination of extended-spectrum β-lactamase-producing *E. coli*. In this study, we report the genome assembly and characterization of a uropathogenic *E. coli* ST405 strain, SZESBLEC201, based on long and short reads obtained from the Nanopore and Illumina sequencing platforms, respectively. Whole-genome sequencing revealed that SZESBLEC201 harbors a 5,020,403 bp chromosome and three plasmids, namely, pSZESBLEC201-1, pSZESBLEC201-2, and pSZESBLEC201-3. pSZESBLEC201-1 (111,621 bp) belongs to the IncFIA-FIB type and harbors *bla*_CTX-M-15_. However, this plasmid does not harbor conjugative transfer-associated genes, rendering pSZESBLEC201-1 unable to be conjugatively transferred. pSZESBLEC201-2 (95,138 bp) is a phage-like plasmid that shows a strong genome synteny with *Escherichia* phage P1 but with the absence of mobile genetic elements and some regulatory genes. pSZESBLEC201-3 (92,865 bp) belongs to the IncI1 type and carries *bla*_CTX-M-24_. In contrast to pSZESBLEC201-1, pSZESBLEC201-3 retains its full active conjugation machinery and can be transferred *via* conjugation. The genetic features of the genome show that the SZESBLEC201 has a unique virulence pattern compared with genetically similar strains found in the same country (China). The plasmid backbones exhibit a high degree of similarity to those of geographically distant isolates, highlighting the global spread of *bla*_CTX-M_ genes and the genome plasticity of this clonal group. The coexistence of two *bla*_CTX-M_ variants in the same strain increases the risk of the emergence of new *bla*_CTX-M_ variants. Further studies on phage-like plasmids are necessary to provide insights into their biological activities and clinical significance.

## Introduction

Uropathogenic *Escherichia coli* (UPEC) is the predominant etiological agent in urinary tract infections (UTIs), accounting for 75–85% of uncomplicated UTI cases globally (Flores-Mireles et al., 2015). The 2016–2017 Study for Monitoring Antimicrobial Resistance Trends in China revealed that UPEC accounted for 60.5% of all UTI cases in the country; 59.0% of these cases (hospital acquired) and 64.3% (community acquired) were caused by extended-spectrum β-lactamase (ESBL) producers (Zhang et al., 2019). ESBLs are the enzymes capable of hydrolyzing penicillin, cephalosporins, and monocyclic amide antibiotics, thus limiting treatment options (Bush and Bradford, 2019). Over a dozen ESBL family proteins have been identified, with *bla*_CTX-M_ being the most prevalent. CTX-M-type β-lactamases are grouped into six sublineages based on amino acid sequence similarity: *bla*_CTX-M_ Group 1, *bla*_CTX-M_ Group 2, *bla*_CTX-M_ Group 8, *bla*_CTX-M_ Group 9, *bla*_CTX-M_ Group 25, and the *Kluyvera cryocrescens* (KLUC) group (Mathers et al., 2015). The distribution of *bla*_CTX-M_ variants in China (both hospital and community isolates) differs from those in most countries, where *bla*_CTX-M-15_ is predominant (Bevan et al., 2017). The nationwide surveillance program in China in 2011-2012 reported that the *bla*_CTX-M-14_ variant belonging to *bla*_CTX-M_ Group 9 was predominant in all *bla*_CTX-M_-positive UPEC isolates (37%), followed by the *bla*_CTX-M-55_ (20%) and *bla*_CTX-M-15_ (14%) variants, both of which belong to *bla*_CTX-M_ Group 1 (Xia et al., 2014). Similar prevalence patterns of *bla*_CTX-M_ have also been observed in *E. coli* strains isolated from food animals and community-acquired urinary tract, respiratory, wound, and blood infections in China (Rao et al., 2014; Zhang et al., 2014). These findings suggest a cross-sectoral transmission of ESBL-producing *E. coli* from food animals to humans. The transmission routes could be through direct contact with colonized animals, their body fluid, or excreta or through the consumption of contaminated food (Zhang et al., 2014). One study has shown that identical plasmid-bearing ESBL genes were present in genetically unrelated human and animal *E. coli* isolates (de Been et al., 2014). These findings indicate that the horizontal gene transfer of ESBLs *via* the dissemination of mobile genetic elements among animal and human isolates plays a greater role than the clonal spread of bacterial isolates (de Been et al., 2014). CTX-M-type ESBLs are associated with international multidrug-resistant (MDR) high-risk clones such as *E. coli* sequence-type (ST) clonal groups 131, 38, and 405 (Mathers et al., 2015). *E. coli* ST405 is the most prevalent clonal group among *bla*_CTX-M-15_-carrying *E. coli* isolates (Xia et al., 2014).

Compared with other pathogenic *E. coli*, a higher proportion of UPEC isolates from China harbor two *bla*_CTX-M_ groups. The most common *bla*_CTX-M_ combinations coexisting in a single UPEC isolate are *bla*_CTX-M-55_ and *bla*_CTX-M-14_ or *bla*_CTX-M-15_ and *bla*_CTX-M-14_ (Xia et al., 2014). However, the reports of *bla*_*CTX*-M-15_ and *bla*_CTX-M-24_ variants coexisting in the same strain are scarce. We recently isolated MDR *E. coli* ST405 from a patient suffering from a UTI. The strain harbored a *bla*_CTX-M-15_-encoding IncFIA-FIB plasmid, a *bla*_CTX-M-24_-encoding IncI1 plasmid, and a phage-like plasmid. ST405 is a common extraintestinal pathogenic *E. coli* clone associated with the global spread of ESBLs, most notably the *bla*_CTX-M_ groups (Peirano and Pitout, 2019). *E. coli* ST405 has recently caused several outbreaks in China (Li et al., 2021), raising concerns about its public health threat. Understanding the mechanisms underlying its increasing ability to cause outbreaks requires an in-depth knowledge of the genetic features of ST405. *bla*_CTX-M-24_ has been found in IncP1 (Hounmanou et al., 2021), IncFII (Yang et al., 2020), IncH1B-FIB (Shen et al., 2019), and IncFI plasmids (Nguyen et al., 2010). However, there are no complete sequences of IncI1 plasmids carrying *bla*_CTX-M-24_ in publicly available genome databases. *bla*_CTX-M-15_ predominates in IncI1-type plasmids, followed by *bla*_*CTX-M-*1_ (Carattoli et al., 2021). To understand the antibiotic resistance dissemination potential of SZESBLEC201, we characterized its genome based on long and short reads obtained from the Nanopore and Illumina sequencing platforms, respectively.

## Materials and Methods

### Bacterial Isolate and Antimicrobial Susceptibility Testing

The MDR *E. coli* isolate SZESBLEC201 was recovered from a urine specimen of a patient suffering from a UTI on the fourth day of hospitalization. The minimum inhibitory concentrations (MICs) of ampicillin, amoxicillin/clavulanic acid, amikacin, aztreonam, chloramphenicol, ceftazidime, ciprofloxacin, cefotaxime, cefazolin, cefepime, gentamicin, imipenem, levofloxacin, meropenem, piperacillin, ampicillin/sulbactam, trimethoprim/sulfamethoxazole, tetracycline, and piperacillin/tazobactam were determined by the microdilution method. The inhibition zone diameter of cefoperazone/sulbactam was determined by the Kirby-Bauer disk susceptibility test, and the breakpoints were interpreted according to the manufacturer’s recommendations (Bio-Rad, Marnes-la-Coquette, France). *E. coli* reference strain ATCC25922 was used as quality control. The MIC values were interpreted according to the Clinical and Laboratory Standard Institute’s Performance Standards for Antimicrobial Susceptibility Testing 29th Edition.

### Conjugative Mating, S1-PFGE, and Southern Blot Hybridization

The conjugative transferability of plasmids in SZESBLEC201 was investigated by solid mating conjugation as previously described (Rodriguez-Grande and Fernandez-Lopez, 2020). *bla*_CTX-M_-producing SZESBLEC201 and *E. coli* J53 strains served as a *bla*_CTX-M_ donor and recipient for the conjugative mating pair, respectively. The mating inoculum was diluted and spread onto four Luria-Bertani agar plates supplemented with 3.4 mM sodium azide and 8 μg/ml cefotaxime for transconjugant selection. After incubation at 37°C overnight, three colonies were randomly selected from each selective agar plate for subsequent S1-nuclease digestion followed by pulsed-field gel electrophoresis (PFGE) as previously described (Barton et al., 1995). Southern blot hybridization was performed, and the presence of plasmids carrying *bla*_CTX-M_ Group 1 and *bla*_CTX-M_ Group 9 genes was detected using digoxigenin-labeled probes (Saladin et al., 2002). FIB replicons in plasmids were also detected using a RepFIB specific probe derived from a PCR-based replicon-typing scheme (Carattoli et al., 2005). The primer pairs used to synthesize the hybridization probes are listed in Supplementary Table 1.

### DNA Purification and Whole-Genome Sequencing

Total DNA was purified from overnight culture using Qiagen Genomic-tip 100/G columns (Qiagen, Germantown, MD, United States) per the manufacturer’s instructions. Whole-genome sequencing (WGS) was performed on the Illumina HiSeq 2500 platform and the Oxford MinIon Nanopore platform. FastQC (version 0.11.9)^1^ and NanoQC (Version 0.9.4)^2^ were used to assess the quality of short reads generated by Illumina and long reads generated by MinIon, respectively. High-quality long reads were assembled *de novo* using Canu (version 2.1.1)^3^ (Koren et al., 2017). Contigs were circularized by Circlator^4^ using the following parameters: merge_min_id, 85; merge_breaklen, 1,000; bwa_opts, -x ont2d; assembler, canu (Hunt et al., 2015). High-quality short reads were used to correct circularized contigs with two iterations of Pilon (version 1.24)^5^ (Walker et al., 2014) correction and one round of Racon (version 1.4.3)^6^ (Vaser et al., 2017) polishing. All programs were run with default parameters unless otherwise specified.

### Bioinformatic Analysis

Gene predictions were performed using Glimmer (version 3.02b)^7^ (Delcher et al., 2007). Gene features and functions were manually annotated using the following databases: RAST tool kit on the Pathosystems Resource Integration Center (PATRIC) Bioinformatics Resource Center^8^ (Davis et al., 2020), Gene Ontology Resource,^9^ Database of Clusters of Orthologous Genes,^10^ and Kyoto Encyclopedia of Genes and Genomes.^11^ Multilocus sequence typing (MLST), plasmid MLST (pMLST), acquired resistance genes, acquired virulence factors, and plasmid incompatibility replicon types (Inc.) were identified from the Center for Genomic Epidemiology website^12^ (Larsen et al., 2012; Zankari et al., 2012; Carattoli et al., 2014; Joensen et al., 2014). Insertion sequences were identified by using ISfinder^13^ (Siguier et al., 2006). The genetic features presented as a circular map of the chromosome were created using PATRIC. The closest relatives of the SZESBLEC201 genome in China were identified using Mash/MinHash in the PATRIC Similar Genome Finder function (Ondov et al., 2016). The multiple-sequence alignment of single-copy orthologous genes shared by all similar genomes was performed using MAFFT version 7^14^ (Katoh and Standley, 2013). Aligned sequences were concatenated to generate whole-genome alignment for subsequent maximum likelihood phylogenetic tree construction by FastTree^15^ (Price et al., 2009). The PATRIC Similar Genome Finder and BLASTn were used to search for possible homologous plasmid sequences (Ondov et al., 2016). The five plasmids with the highest bit score of the query plasmids were retrieved for subsequent comparative analyses. The genetic features presented as circular maps of each plasmid were obtained using the CGView server.^16^ A comparative analysis of plasmids was performed using BLAST Ring Generator version 0.95^17^ (Alikhan et al., 2011) and Easyfig (version 2.2.3)^18^ (Sullivan et al., 2011). The Bayesian Markov chain Monte Carlo method implemented in the BEAST2 package was used to identify plasmid evolution over time with the GTR-I substitution model (Bouckaert et al., 2014).

## Results

### Antimicrobial Resistance Profile

The SZESBLEC201 isolate was resistant to ampicillin, aztreonam, ceftazidime, ciprofloxacin, cefotaxime, cefazolin, cefepime, gentamicin, levofloxacin, piperacillin, trimethoprim/sulfamethoxazole, and tetracycline. It was susceptible to amoxicillin/clavulanic acid, amikacin, chloramphenicol, imipenem, meropenem, ampicillin/sulbactam, cefoperazone/sulbactam, and piperacillin/tazobactam (Supplementary Table 2).

### Conjugative Mating, S1-PFGE, and Southern Blot Hybridization

**FIGURE 1:**
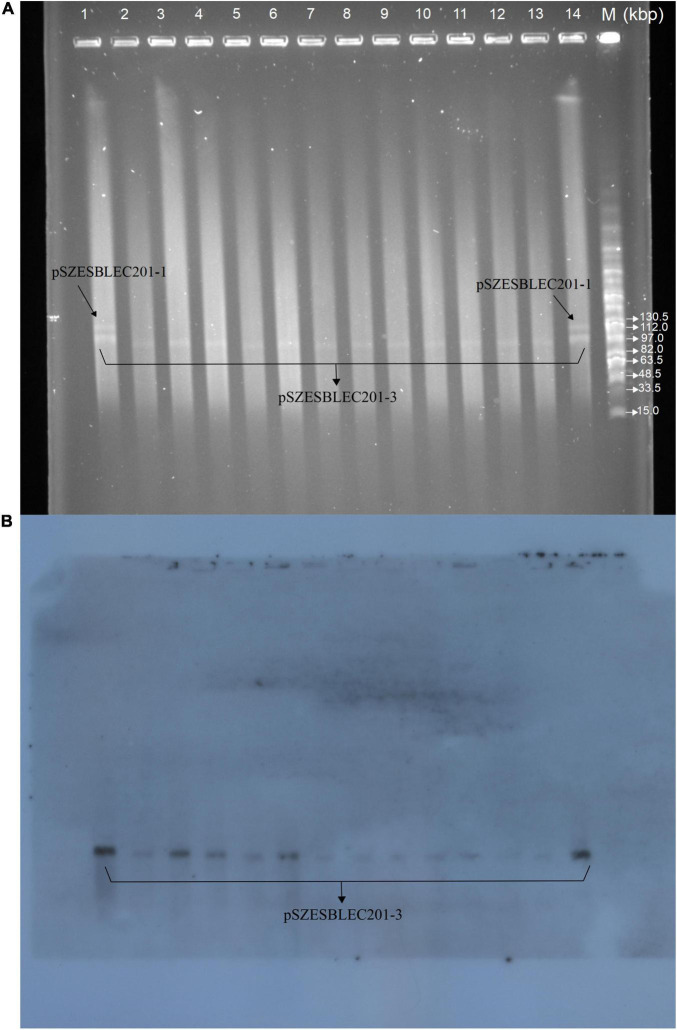
S1-PFGE plasmid pattern and Southern blot for strain SZESBLEC201 and transconjugant TcJ53-CTX-M. **(A)** PFGE result for S1-digested plasmids of strain SZESBLEC201. Lanes 1 and 14: plasmid profile of strain SZESBLEC201, Lanes 2–13: plasmid profile of transconjugant TcJ53-CTX-M. Lane M: New England Biolabs MidRange PFG marker. **(B)** Southern blot hybridization with probe specific to *bla*_CTX-M_ Group 9.

S1-PFGE showed that SZESBLEC201 harbored two plasmids, which were approximately 110 and 90 kbp in size (Figure 1A). WGS analysis identified a third plasmid. Randomly selected transconjugant TcJ53-CTX-M acquired the 90 kbp plasmid from donor SZESBLEC201, confirming the conjugative transferability of the plasmid. Transconjugant TcJ53-CTX-M inherited the 3rd and 4th cephalosporin-resistant traits from donor SZESBLEC201 after the acquisition of the *bla*_CTX-M_-bearing plasmid. Southern blot hybridization indicated that the *bla*_CTX-M_ gene belonged to the Group 9 sublineage and was located on the 90 kbp plasmid (Figure 1B), whereas the *bla*_CTX-M_ gene belonged to the Group 1 sublineage and was located on the 110 kbp plasmid (Supplementary Figure 1).

### General Genomic Features of the SZESBLEC Isolate

**FIGURE 2:**
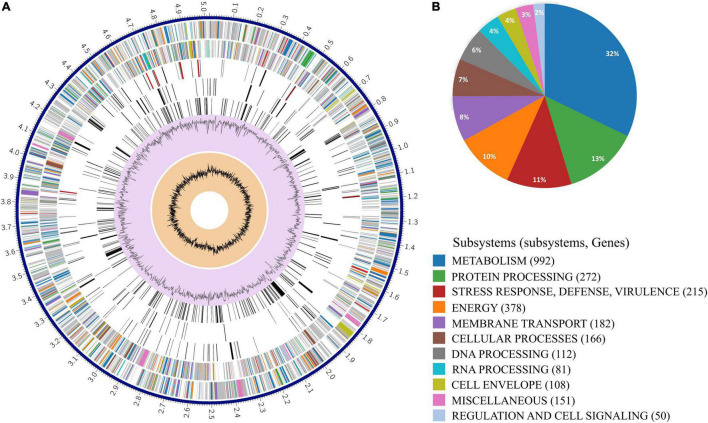
Circular representation of strain SZESBLEC201 chromosome. **(A)** From the outer to inner rings: the assembled contig, forward strand CDSs, reverse strand CDSs, RNA genes, CDSs with homology to known transporters, CDSs with homology to known virulence factors, genome GC content, and distribution of the GC skew. Colors of the CDSs on the forward and reverse strand indicate the subsystems that these genes belong to. **(B)** Proportions of each subsystem in the strain SZESBLEC201 chromosome.

The final assembled and circularized SZESBLEC201 genome consisted of 5,320,027 bases (Supplementary Table 3). Whole-genome MLST revealed that SZESBLEC201 belongs to ST405. ResFinder found that the SZESBLEC201 chromosome carries a multidrug transporter *mdf*A gene. The ciprofloxacin resistance was due to the mutations in the chromosomally located *gyr*A gene (substitutions S83L and D87N), *par*C gene (substitution S80I), and *par*E gene (substitution S458A). The chromosome of SZESBLEC201 had a GC content of 50.79%, and this genome had 5,114 coding sequences (CDSs), 85 transfer RNA (tRNA) genes, and 22 ribosomal RNA genes. A total of 2,724 genes were classified into 12 subsystems based on their specific biological process or structural complex (Figure 2). The largest percentage of these genes were metabolism-associated genes (37%), followed by energy-associated genes (14%), and protein processing genes (10%).

WGS analysis revealed three plasmids present in the SZESBLEC201 genome, designated as pSZESBLEC201-1 (111, 621 bp), pSZESBLEC201-2 (95,138 bp), and pSZESBLEC201-3 (92,965 bp) (Supplementary Table 3). pSZESBLEC201-2 and pSZESBLEC201-3 were similar in size; they were not resolved in the S1-PFGE and appeared as one band on the agarose gel (Figure 1A). Southern blot hybridization with the RepFIB-specific probe confirmed the presence of a phage-like plasmid (pSZESBLEC201-2) that had a similar size to the *bla*_CTX-M-24_-bearing plasmid (pSZESBLEC201-3) (Supplementary Figure 2). The RepFIB replicon was present in both the *bla*_CTX-M-15_-bearing plasmid (pSZESBLEC201-1) and the phage-like plasmid (pSZESBLEC201-2), but it was absent from the *bla*_CTX-M-24_-bearing plasmid (pSZESBLEC201-3). pSZESBLEC201-1 was found to carry multi-replicons (FAB formula F-:A1:B1) that had a 51.25% GC content. pSZESBLEC201-1 was found to carry multiple resistance genes, including the ESBL-producing *bla*_CTX-M-15_ gene, sulfonamide resistance dihydropteroate synthase *sul*1, the aminoglycoside resistance *aac*(3′)-IIa and *aad*A5 genes, trimethoprim resistance dihydrofolate reductase *dfr*A17, and the tetracycline resistance gene *tet*(B). pSZESBLEC201-2 was a phage-like plasmid with a 48.06% GC content and carried no antibiotic resistance genes. pSZESBLEC201-3 was found to be an IncI1/ST166 plasmid under the pMLST scheme. It had a 49.86% GC content and carried only one resistance-encoding gene, the ESBL-producing *bla*_CTX-M-24_ gene.

### Comparative Genome Analysis of the SZESBLEC201 Isolate

**FIGURE 3:**
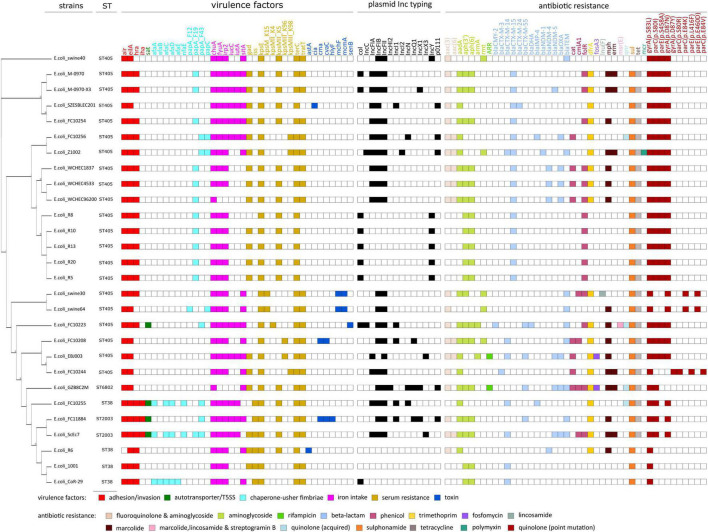
Phylogeny, virulence factors, plasmid profiles, and antibiotic resistance of strain SZESBLEC201 and its genetically similar strains in China. The whole-genome phylogenetic tree was constructed using the maximum likelihood method from concatenated single-copy orthologous genes shared by analyzed genomes. Sequence types, virulence factors, plasmid Inc. typing, and antibiotic resistance were respectively determined by multi-locus sequence typing (MLST), VirulenceFinder, PlasmidFinder, and ResFinder on Center for Genomic Epidemiology.

Twenty-seven *E. coli* strains isolated in China that are genetically closely related to SZESBLEC201 were retrieved by the Mash/MinHash-based Similar Genome Finder in PATRIC (Supplementary Table 4). Among the retrieved strains, 14 strains were isolated from humans, while the others were isolated from animals or sewage. Twenty strains were ST405, four strains were ST38, two strains were ST2003, and one strain was ST6802. A phylogenetic tree based on single-copy orthologous genes showed that SZESBLEC201 was grouped with *E. coli* strain FC10254, sharing 98.86% similarity (Figure 3). The genomes of SZESBLEC201 and the 27 *E. coli* strains were screened with Virulence Finder (Figure 3) to clarify their virulence potential (Joensen et al., 2014). SZESBLEC201 had a unique virulence factor pattern that was not found in the other 27 genetically close related *E. coli* strains. Both autotransporter and chaperone-usher fimbriae were absent, while colicin-Ia (*cia*) was uniquely present in SZESBLEC201. The *cia* gene was located on plasmid pSZESBLEC201-3. This is a channel-forming class of colicin that causes cytoplasmic membrane depolarization, providing a competitive colonization advantage (Waters and Crosa, 1991). Enteroaggregative immunoglobulin repeat protein (*air*), outer membrane hemin receptor (*chu*A), capsule polysaccharide export inner-membrane protein (*kps*E), and tellurium ion resistance protein (*ter*C) were universally present in all analyzed genomes. The plasmid profiles were determined using PlasmidFinder (Carattoli et al., 2014). The results showed that the plasmid profiles of the 28 analyzed genomes are diverse, although identical plasmid profiles are shared by strains isolated from the same collection center (Figure 3 and Supplementary Table 4). The *E. coli* GZB8C2M genome contained the greatest number of replicons identified under the PlasmidFinder typing scheme. IncF-type plasmids (including IncFIA, IncFIB, and IncFII) were the most abundant, being identified in 20 of the analyzed genomes. The second most abundant plasmid was the col-like plasmid, being identified in nine analyzed genomes. The p0111-like plasmid was found in five of the 28 analyzed genomes. No plasmid profiles similar to that of SZESBLEC201 were identified. There were three sets of identical plasmid Inc profiles found in the analyzed genomes (Figure 3). The first set (strains WCHEC1837, WCHEC4533, and WCHEC96200) consisted of FIA, FIB, and FII replicons, the second set (strains R8, R10, R13, R20, and R5) consisted of col-like and IncY replicons, and the third set (strains swine30 and swine 64) consisted of FIB and FII replicons. The plasmid sequences of WCHEC1837, WCHEC4533, and WCHEC96200 were retrieved from GenBank for comparison and found the IncFIA-FIB-FII-type plasmid harboring *bla*_CTX-M-15_ overlapped in the three strains (data not shown). The other strains showing identical plasmid profiles neither had raw sequencing data available for the reconstruction of plasmid sequences, nor were complete plasmid sequences deposited, precluding the identification of other overlapped plasmids within the selected genomes.

The presence of antibiotic resistance genes in the analyzed genomes was also investigated using ResFinder (Zankari et al., 2012). Thirty-four acquired antibiotic resistance genes were identified (Figure 3), with an average of nine genes per genome. Aminoglycoside resistance-associated and ESBL-producing genes were present in all 28 analyzed genomes, indicating genotypic resistance against aminoglycoside and β-lactam antibiotics. In addition to SZESBLEC201, the genomes of *E. coli* Z1002 and EBJ2003 harbored more than one *bla*_CTX-M_ variant. *bla*_CTX-M-14_ and *bla*_CTX-M-15_ were found in the genome of Z1002, whereas *bla*_CTX-M-14_ and *bla*_CTX-M-55_ were found in the genome of EBJ2003. Similar to the SZESBLEC201 genome, different *bla*_CTX-M_ variants in the genomes of Z1002 and EBJ2003 are located on separate plasmids. S83L substitution in *gyr*A was present in all analyzed genomes. Double substitution (S83L and D87N) in *gyr*A was found in 21 genomes, among which, 18 were ST405 strains. S80I substitution in *par*C was found in 22 genomes, 18 of which belonged to ST405. S458A substitution in *par*E was also found in the same 18 ST405 strains.

### Comparative Analysis of pSZESBLEC201-1

**FIGURE 4:**
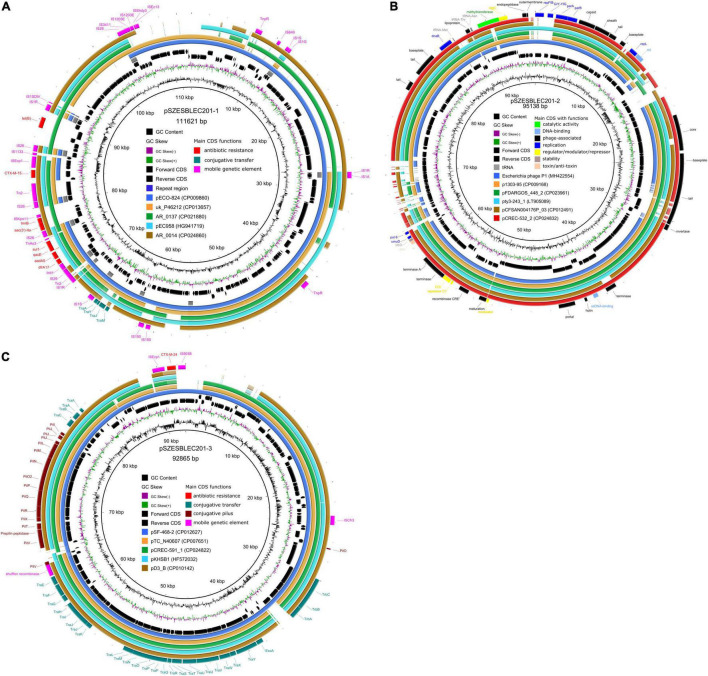
Comparative schematic representation of pSZESBLEC201-1, pSZESBLEC201-2, and pSZESBLEC201-3 with their closest related plasmids. **(A)** Multiple genome comparison using BLAST ring image generator (BRIG) with pSZESBLEC201-1 as the reference. **(B)** Multiple genome comparison using BRIG with pSZESBLEC201-2 as the reference. **(C)** Multiple genome comparison using BRIG with pSZESBLEC201-3 as the reference.

**FIGURE 5:**
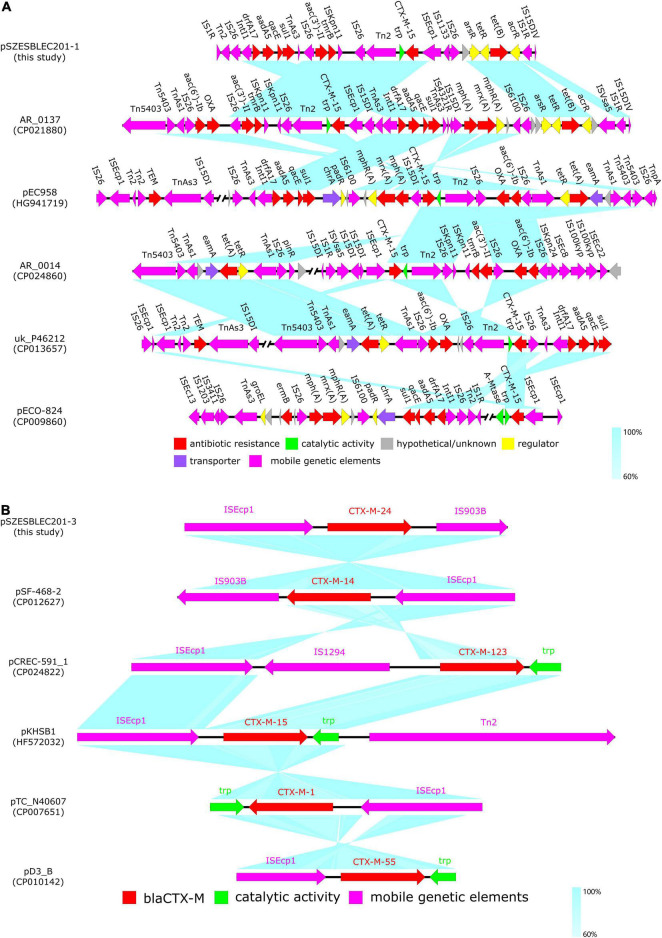
Linear comparison of pSZESBLEC201-1 and pSZESBLEC201-3 resistance regions with those of their closest related plasmids. **(A)** Comparison of pSZESBLEC201-1 MDR region with those of plasmids AR_0137, pEC958, AR_0014, uk_P46212, and pECO-824. **(B)** Comparison of the genetic environment surrounding *bla*_CTX-M_ in pSZESBLEC201-3, pSF-468-2, pCREC-591_1, pKHSB1, pTC_N40607, and pD3_B. Open reading frames are indicated with arrows. Homologous regions in different plasmids are linked with turquoise coloring, and degrees of homology are denoted by color depth. Red, bright green, gray, yellow, purple, and fuchsia represent genes associated with antibiotic resistance, catalytic activity, hypothetical proteins, regulation, transportation, and mobile genetic elements, respectively.

The similar Genome Finder search in PATRIC showed that pSZESBLEC201-1 had the closest relatedness (81% query coverage and 99.98% identity) with a previously reported plasmid, pECO-824 (accession no. CP009860), which was isolated from the carbapenemase-producing *E. coli* strain ECONIH1 in the United States (Conlan et al., 2014). The other similar plasmids identified were uk_P946212 (73% query coverage, 99.97% identity) from *E. coli* strain uk_P46212 isolated in the United Kingdom, AR_0137 (76% query coverage, 99.92% identity) from *E. coli* strain AR_0137 isolated in the United States, pEC958 (65% query coverage, 99.23% identity) from *E. coli* strain EC958 isolated in Australia, and AR_0014 (71% query coverage, 99.92% identity) from *E. coli* strain AR_0014 isolated in the United States (Figure 4A and Supplementary Figure 3). pSZESBLEC201-1 and pECO-824 shared an ∼80 kbp backbone containing the majority of genes responsible for replication, immunity, virulence, regulation, metabolism, plasmid stability, and transportation. However, the two plasmids possessed markedly varied resistance regions (Figure 5A). Resistance genes *dfr*A17, *aad*A5, *sul*1, *aac*(3′)-IIa, *tmr*B, *bla*_CTX-M-15_, and *tet*(B) were located in the position of the 73–90 kbp region of pSZESBLEC201-1. By contrast, pECO-824 possessed two resistance regions, *mph*(A), *sul*1, and *aad*A5 were located in a 10 kbp-sized MDR region, and there was a *bla*_CTX-M-15_ region located ∼50 kbp downstream of the MDR region. The MDR region of plasmid AR_0137, which carried resistance genes *aac*(6′)-Ib, *bla*_*OXA*_, *aac*(3′)-IIa, *tmr*B, *bla*_CTX-M-15_, *drf*A17, *aad*A5, *sul*1, *mph*(A), and *tet*(B), exhibited the highest degree of homology (76% query coverage and 99.76% identity) with that of pSZESBLEC201-1 among all similar plasmids. A significant number of conjugative transfer-associated genes present in the five plasmids most similar to pSZESBLEC201-1 were not present in pSZESBLEC201-1 itself (Supplementary Figure 3). This finding was consistent with the conjugation experiment result, indicating that this plasmid is not conjugatively transferable.

The genetic environment of the *bla*_CTX-M-15_ structure in pSZESBLEC201-1 was compared with those of the five plasmids with high pSZESBLEC201-1 similarity retrieved from PATRIC (Figure 5A). In pSZESBLEC201-1, AR_0137, and AR_0014, the *bla*_CTX-M-15_ genes were found to be located within the same genetic contexts. Either a complete or partial insertion sequence IS*Ecp1* was found upstream and in the same orientation as the *bla*_CTX-M-15_ genes and also at the same distances from the *bla*_CTX-M-15_ genes; the tryptophan synthase *trp* proximally flanked the *bla*_CTX-M-15_ genes downstream with the transposase Tn*2* and insertion sequence IS*26* being located further, forming an IS*Ecp1*–*bla_CTX-M-15_*–*trp*–Tn*2*–IS*26* unit. pEC958 and uk_P46212 had identical genetic contexts downstream of the *bla*_CTX-M-15_ genes to those of pSZESBLEC201-1, AR_0137, and AR_0014 but varied in the genes upstream of the *bla*_CTX-M-15_. IS*15DI* and IS*26* was found located upstream of the *bla*_CTX-M-15_ genes in pEC958 and uk_P46212, respectively. In the case of pECO-824, the *bla*_CTX-M-15_ was flanked by the IS*Ecp1* upstream and the *trp* downstream, but the adenine-specific methyltransferase *A-Mtase* was found located proximal to *trp*, forming an IS*Ecp1*–*bla*_CTX-M-15_–*trp*–*A-Mtase* unit.

### Comparative Analysis of pSZESBLEC201-2

A comparative analysis was performed with the chosen reference genome *Escherichia* phage P1 (accession no. MH42254). pSZESBLEC201-2 was found to have a strong nucleotide sequence identity with the chosen P1 reference genome (86% query coverage, 97.61% identity) (Figure 4B and Supplementary Figure 4). The replication region of pSZESBEC201-2 corresponded to the phage P1 replicons (including RepFIB, GIY-YIG nuclease, *par*A, and *par*B) and flanking regions, sharing 100% nucleotide identities. Three major regions, including two copies of truncated transposase Tn*As1*, a TonB-dependent receptor upstream of the modulator, and the transcription regulator *lex*A upstream of error-prone repair gene *umu*D, were missing in pSZESBLEC201-2 but were present in the P1 reference genome (Supplementary Figure 4).

The five plasmid sequences that were most highly homologous to pSZESBLEC201-2 were identified using the Similar Genome Finder on PATRIC and BLAST searches. The plasmid sequences were retrieved from the GenBank database for comparison with pSZESBLEC201-2. These plasmid sequences included one from *Salmonella enterica* (pty3-243_1) and four from *E. coli* (p1303_95, pFDAARGOS_448_2, pCFSAN004176P_03, and pCREC-532_2). The level of homology to the pSZESBLEC201-2 sequence is shown in Figure 4B and Supplementary Figure 4. pSZESBLEC201-2 shared 94 of 122 CDSs with its similar plasmids, of which 79 CDSs had 95–100% nucleotide sequence identity. In addition, 64 phage-related genes of pSZESBLEC201-2 had more than 90% nucleotide sequence identity with p1303_95. These phage-related genes in pSZESBLEC201-2 included the tail, tail fiber, core, holin, portal baseplate, invertase, terminase, and outer membrane lipoprotein. The three tRNAs (tRNA-Met, tRNA-Thr, and tRNA-Asn) present in pSZESBLEC201-2 were identical to those present in its similar plasmids. Plasmid partitioning genes *par*A and *par*B, replicon stabilization gene *stb*, and phage replicon *rep*L were found in all analyzed plasmids with high degrees of nucleotide similarity. Although RepFIB genes were found in all analyzed plasmids, RepFIB in pSZESBLEC201-2 had less than 50% nucleotide identity with those of other plasmids, except for pty3-243_1 (98% nucleotide identity).

### Comparative Analysis of pSZESBLEC201-3

The comparative analysis of pSZESBLEC201-3 was performed by comparing it with five highly homologous plasmid sequences, including pSF-468-2 (accession no. CP012627), pTC_N46067 (accession no. CP007651), pCREC-591_1 (accession no. CP024822), pKHSB1 (accession no. HF572032), and pD3_B (accession no. CP010142) (Figure 4C and Supplementary Figure 5). All plasmids are harbored by *E. coli*, except for pKSB1, which is harbored by *Shigella sonnei*. In contrast to pSZESBLEC201-1, a major part of the pSZESBLEC201-3 backbone is highly conserved. pSZESBLEC201-3 shared 99.56% identity and 100% coverage with plasmid pSF-468_2, which was harbored by extraintestinal pathogenic *E. coli* ST95 isolated in the United States (Stephens et al., 2015). Both plasmids were composed of an almost identical 90 kbp-sized backbone (Figure 4C and Supplementary Figure 5). The backbone was composed of core genes encoding mating pair formation (*trb*A-C genes), a type IV secretion system (*tra*A-Y genes), shufflon-specific DNA recombinase (*rci*), and conjugative pilus formatting (*pil*I-X) genes.

pSZESBLEC201-3 also exhibited high homology to the other four plasmids (pTC_N40607, pCREC-591_1, pKHSB1, and pD3_B), with 98.68–99.50% identity at 83–92% coverage. These plasmids shared similar plasmid backbones, but the *bla*_CTX-M_ regions varied. In addition, additional resistance-encoding regions were present in the four plasmids but were absent in pSZESBLEC201-3 and pSF-468-2.

The *bla*_CTX-M_ region of pSZESBLEC201-3 was compared with those of its similar plasmids (Figure 5B). Notably, various *bla*_CTX-M_ variants were found on the analyzed plasmids. *bla*_CTX-M-15_, *bla*_CTX-M-1_, and *bla*_CTX-M-55_, belonging to *bla*_CTX-M_ Group 1, were located on pKHSB1, pTC_N40607, and pD3_B, respectively. *bla*_CTX-M-123_, a hybrid of *bla*_CTX-M_ Group 1 and 9 variants (D’Andrea et al., 2013), was located on pCREC-591_1. The *bla*_CTX-M_ regions of pSZESBLEC201-3 and pSF-468-2 were flanked by the IS*Ecp1* upstream and the IS*903B* downstream, resembling a typical IS*Ecp1*–*bla*_CTX-M_-Group 9–IS*903B* structure (Canton et al., 2012); however, the IS*903B* in the *bla*_CTX-M_ region of pSZESBLEC201-3 was truncated. In addition, the two plasmids harbored various *bla*_CTX-M_ Group 9 variants: *bla*_CTX-M-24_ on pSZESBLEC201-3 and *bla*_CTX-M-14_ on pSF-468-2. The pairwise alignment of the two genes revealed that *bla*_CTX-M-24_ and *bla*_CTX-M-14_ had only one nucleotide difference, resulting in an arginine–serine substitution (data not shown). The variations in the IS*Ecp1*–*bla*_CTX-M_-Group 9–IS*903B* structures and in the *bla*_CTX-M_ Group 9 variants suggested that pSZESBLEC201-3 and pSF-468-2 had incorporated the *bla*_CTX-M_ genes from distinct origins. Despite showing high nucleotide similarity, a time-measured phylogeny tree showed that pSZESBLEC201-3 and pSF-468-2 had undergone divergent evolution (Supplementary Figure 6). pD3_B harbored by *E. coli* isolated from dog had the closest evolutionary relation to pSZESBLEC201-3.

## Discussion

In this study, we characterized uropathogenic *E. coli* ST405 strain SZESBLEC201 based on a combination of long-read and short-read sequences. SZESBLEC201 harbored two *bla*_CTX-M_ variants (*bla*_CTX-M-15_ and *bla*_CTX-M-24_), each on a different plasmid, as well as a phage-like plasmid. The coexistence of *bla*_CTX-M_ Group 1 and Group 9 variants in the same isolate has been previously reported in China (Sun et al., 2010; He et al., 2013; Zhang et al., 2014). However, the coexistence of *bla*_CTX-M-15_ and *bla*_CTX-M-24_ has not yet been reported. In SZESBLEC201, *bla*_CTX-M-15_ was found to be located on a non-transferrable IncFIA-FIB plasmid together with multiple antibiotic resistance genes, whereas *bla*_CTX-M-24_ was located on the self-transferrable IncI1 plasmid. Further comparative analysis with plasmid sequences revealed that plasmids highly homologous to the *bla*_CTX-M_-bearing plasmids of the SZESBLEC201 strain are harbored by geographically distant isolates belonging to different sequence types, highlighting the global dissemination of this plasmid-mediated resistance determinant among *E. coli* and other *Enterobacteriaceae* family members (Naseer and Sundsfjord, 2011). In Japan, the positive rates of *bla*_CTX-M-15_ and *bla*_CTX-M-14_ are higher in *E. coli* ST405 than in ST131 (Matsumura et al., 2013). The same study also observed that compared with ST131, a higher proportion of *E. coli* ST405 isolates carried multidrug resistance, raising concerns about the plasticity *E. coli* ST405 acquiring resistance and the potential threat of this clone to public health. Furthermore, the emergence of novel *bla*_CTX-M_ variants is enhanced by recombination events that occur in the same bacterial isolate where two or more *bla*_CTX-M_ variants coexisted (Canton et al., 2012). At least four *bla*_CTX-M_ variants (*bla*_CTX-M-45_, *bla*_CTX-M-64_, *bla*_CTX-M-123_, and *bla*_CTX-M-132_) have been reported to exhibit a hybrid structure of *bla*_CTX-M_ Group 1 and 9 variants (D’Andrea et al., 2013). The homologous recombination between the members of these two *bla*_CTX-M_ groups is probably a driving force of *bla*_CTX-M_ evolution.

The SZESBLEC201 strain demonstrated a susceptibility to conventional β-lactamase inhibitors owing to the absence of carbapenemase-encoding genes and, presumably, a lack of *bla*_CTX-M_ gene overexpression. As yet, no naturally occurring *bla*_CTX-M_-type ESBLs have been found to confer resistance to natural β-lactamase inhibitors (D’Andrea et al., 2013). However, the coexistence of *bla*_CTX-M-15_ and *bla*_OXA-1_ in the plasmids of the IncF group is common (Branger et al., 2018; Livermore et al., 2019). The presence of both *bla*_CTX-M-15_ and *bla*_OXA-1_ considerably reduces the susceptibility to β-lactam/β-lactamase inhibitor combinations. Four plasmids that showed high degrees of similarity to pSZESBLEC201-1 were found to coharbor *bla*_OXA-1_ and *bla*_CTX-M-15_ (AR_0137, pEC958, AR_0014, and uk_P46212) (Figure 5). The *bla*_OXA-1_–*acc*(6′)-Ib–IS*26* elements were present in the four plasmids, suggesting that IS*26* had mediated the insertion into the plasmid backbone. The association between *bla*_OXA-1_ and *aac*(6′)-Ib is strikingly strong. A recent study in the United Kingdom found an extremely high rate (98.7%) of *bla*_OXA-1_ and *acc*(6′)-Ib coexistence among all isolates harboring *aac*(6′)-Ib (Livermore et al., 2019).

The current study highlights the common arrangements of the *bla*_CTX-M-15_ regions that were identified in the IncFIA-FIB plasmids conferring resistance to β-lactams and other widely used classes of antibiotics. It also discusses the structural homogeneity associated with the regions flanking these resistance genes. The insertion sequences IS*Ecp1* and IS*26* are common in the genetic elements surrounding *bla*_CTX-M_ genes (Zhao and Hu, 2013). IS*Ecp1* contributes to the transcription, expression, and mobilization of *bla*_CTX-M_ genes (Poirel et al., 2012; Zhao and Hu, 2013). IS*Ecp1* can capture other resistance genes by incorporating the regions of gene cassettes, increasing the risk of coselection (Partridge, 2011).

A significant degree of conjugation gene loss was observed in the *bla*_CTX-M-15_-bearing IncFIA-FIB plasmid pSZESBLEC201-1. Active conjugation machinery possibly increases the bacterial fitness burden, and conjugation-associated *tra* genes were possibly lost during the vertical transfer of the plasmid (Phan et al., 2015). In contrast, *bla*_CTX-M-24_-bearing IncI1 plasmid pSZESBLEC201-3 retained its full active conjugation machinery. IncI1 plasmids are featured with two types of conjugative pili (Sampei et al., 2010). The *tra* and *trb* genes encode thick conjugative pili, while the *pil* genes encode thin flexible pili, enabling the conjugative transfer of IncI1 plasmid in solid and liquid environments (Yoshida et al., 1999; Dudley et al., 2006).

In addition to the two *bla*_CTX-M_-bearing plasmids in the SZESBLEC201 genome, a phage-like plasmid, pSZESBLEC201-2, was also present. This plasmid showed strong genome synteny with *Escherichia* phage P1 with almost identical genes responsible for replication and stability but with the absence of mobile genetic elements and several regulatory genes. *Escherichia* phage P1 can infect and lysogenize host *E. coli* cells and persist within the host cell as a free circular plasmid (Lobocka et al., 2004). No clear boundaries exist between the P1 temperate phage and a phage-like plasmid. Plasmids are usually typed according to their incompatibility with other plasmids, and temperate phages are classified according to the genomic relatedness of the virion structure (Venturini et al., 2019). Phage-like plasmids can be classified either by phage taxonomy or plasmid incompatibility because virion and plasmid replicons are both present. pSZESBLEC201-2 consisted of a large portion of phage structure-encoding genes and the RepFIB replicon; therefore, it was classified as a phage-like plasmid. This plasmid could not be typed using PlasmidFinder’s Inc. typing scheme, but it was identified as a p0111-like plasmid. This classification is expected because of the presence of more phage-associated genes than plasmid-associated genes in the pSZESBLEC201-3 genome, and the PlasmidFinder database is skewed towards plasmids that are commonly found in *Enterobacteriaceae* (Carattoli et al., 2014). Less effort has been devoted to phage-like plasmids because they carry genes that lack clinical and therapeutic values. However, resistance gene-bearing phage-like plasmids have been documented in recent years (Xu et al., 2018; Ballaben et al., 2019; Galetti et al., 2019). Additional effort with the aid of new sequencing technologies is necessary to gain insights into the roles of phage-like plasmids in the dissemination of antibiotic resistance.

## Conclusion

Although the coexistence of two or more *bla*_CTX-M_ variants in the same strain has been reported elsewhere, this is the first study to report *E. coli* ST405 harboring *bla*_CTX-M-15_ and *bla*_CTX-M-24_ variants in the same isolate in China. This study describes the characteristics of the uropathogenic *E. coli* strain SZESBLEC201 genome and presents a comparative analysis of the genome and *bla*_CTX-M_-bearing plasmids. This strain possesses unique patterns of virulence factors in comparison with its closely related strains isolated within the same country, and its *bla*_CTX-M_-bearing plasmids are more related to those in geographically distant *E. coli* isolates. The availability of the complete genome of *E. coli* ST405 facilitates a further investigation of the underlying mechanism of becoming a globally successful clone and the potential roles played by its *bla*_CTX-M_-bearing plasmids and phage-like plasmid in gene transfer.

## Data Availability Statement

The data presented in the study have been deposited in the GenBank repository under accession numbers CP090074-CP090077.

## Author Contributions

MY and QZ performed the conjugation experiment, Illumina platform-based whole-genome sequencing, and bioinformatic analysis and drafted the manuscript. JZ and AS performed the bacterial isolation, antimicrobial susceptibility testing, nucleic acid purification, and PCR. SH performed the Nanopore platform-based whole-genome sequencing. JQ, ZH, and PL conceived the study, designed the experiments, and reviewed the manuscript. All authors read and approved the manuscript.

## Conflict of Interest

The authors declare that the research was conducted in the absence of any commercial or financial relationships that could be construed as a potential conflict of interest.

## Publisher’s Note

All claims expressed in this article are solely those of the authors and do not necessarily represent those of their affiliated organizations, or those of the publisher, the editors and the reviewers. Any product that may be evaluated in this article, or claim that may be made by its manufacturer, is not guaranteed or endorsed by the publisher.

## Footnotes

1
https://github.com/s-andrews/FastQC

2
https://github.com/wdecoster/nanoQC

3
https://github.com/marbl/canu

4
https://github.com/sanger-pathogens/circlator

5
https://github.com/broadinstitute/pilon

6
https://github.com/isovic/racon

7
http://ccb.jhu.edu/software/glimmer/index.shtml

8
https://www.patricbrc.org/

9
http://www.geneontology.org

10
https://www.ncbi.nlm.nih.gov/research/cog

11
https://www.genome.jp/kegg/

12
https://www.genomicepidemiology.org/

13
https://isfinder.biotoul.fr/

14
https://mafft.cbrc.jp/alignment/software/

15
http://www.microbesonline.org/fasttree/

16
http://cgview.ca/

17
http://brig.sourceforge.net/

18
https://mjsull.github.io/Easyfig/
