# Supplementary figures and images for: Genomic Characterization of a Uropathogenic Escherichia coli ST405 Isolate Harboring blaCTX-M-15-Encoding IncFIA-FIB Plasmid, blaCTX-M-24-Encoding IncI1 Plasmid, and Phage-Like Plasmid
Source: Front Microbiol. 2022 Apr 11;13:845045. doi: 10.3389/fmicb.2022.845045 (PMC9037040; doi:10.3389/fmicb.2022.845045)

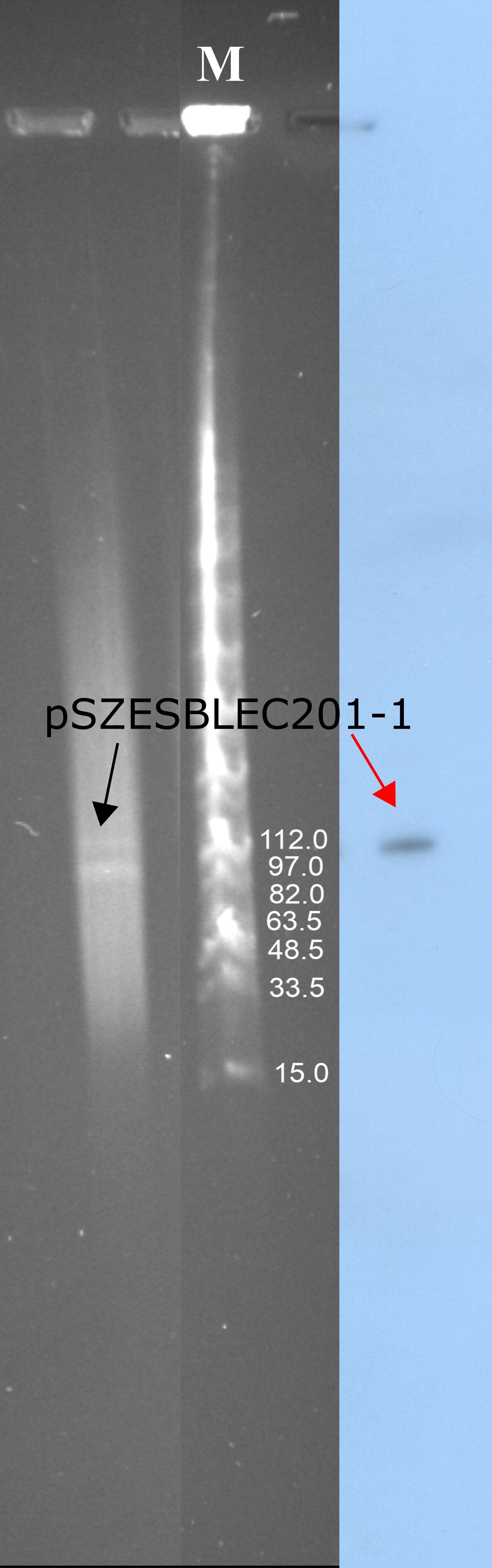

Supplement: Supplementary file 1 [file Image_1.JPEG]

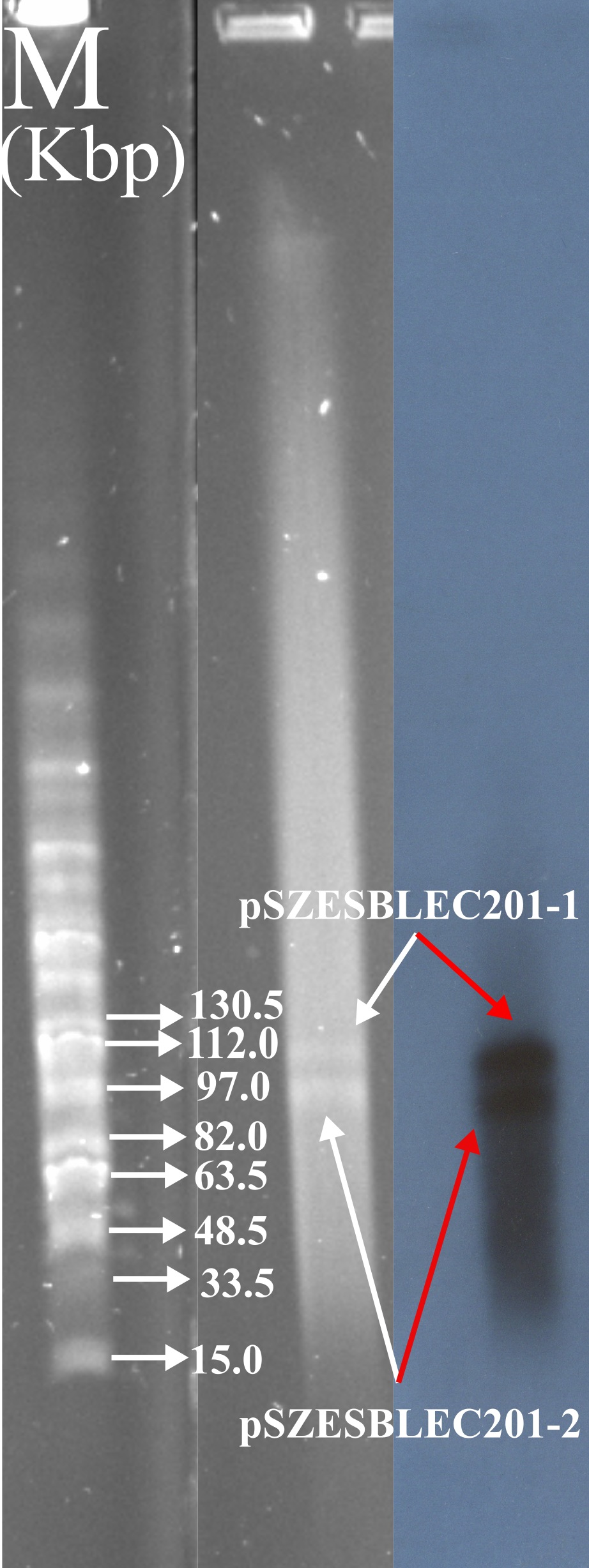

Supplement: Supplementary file 2 [file Image_2.JPEG]

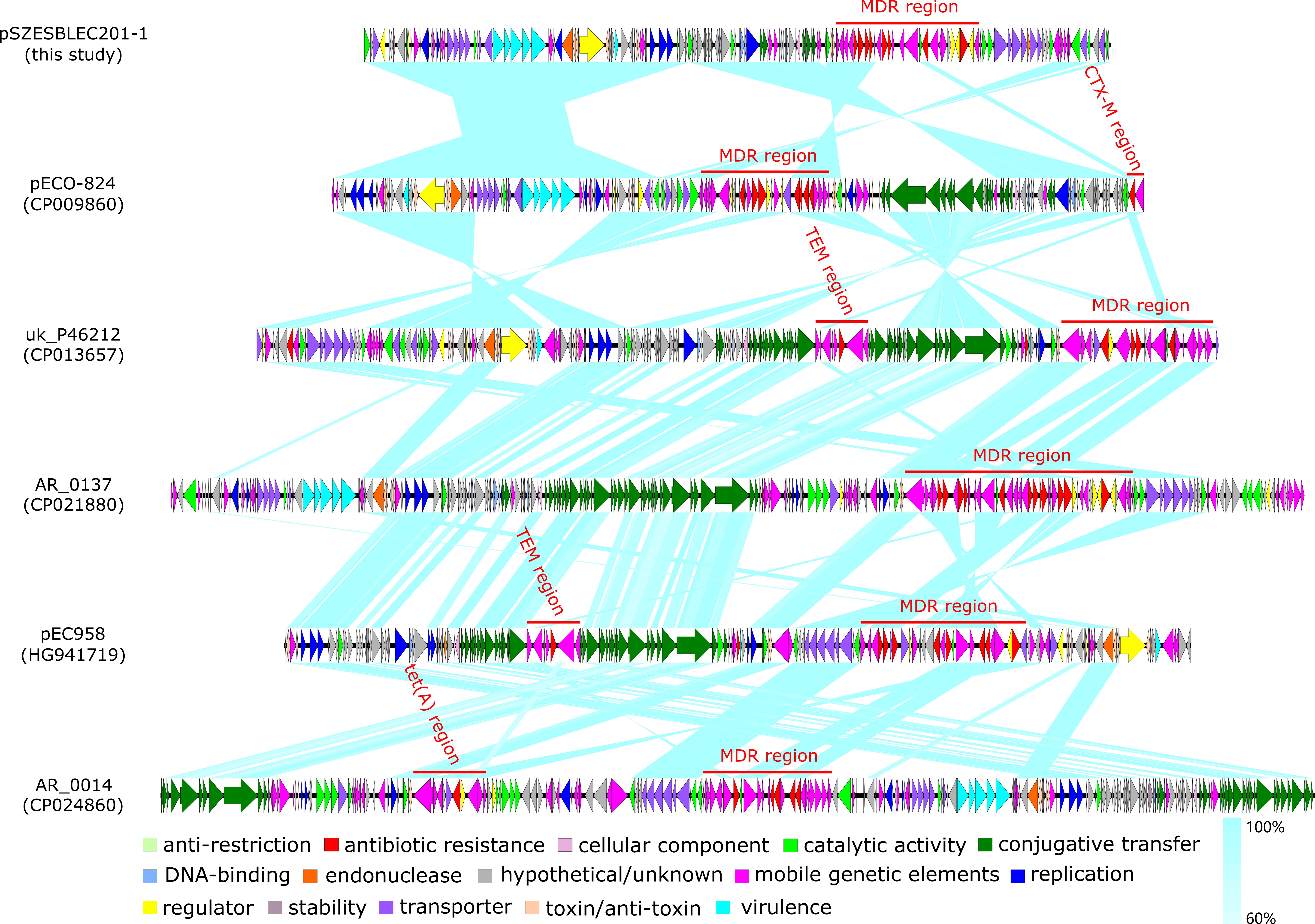

Supplement: Supplementary file 3 [file Image_3.JPEG]

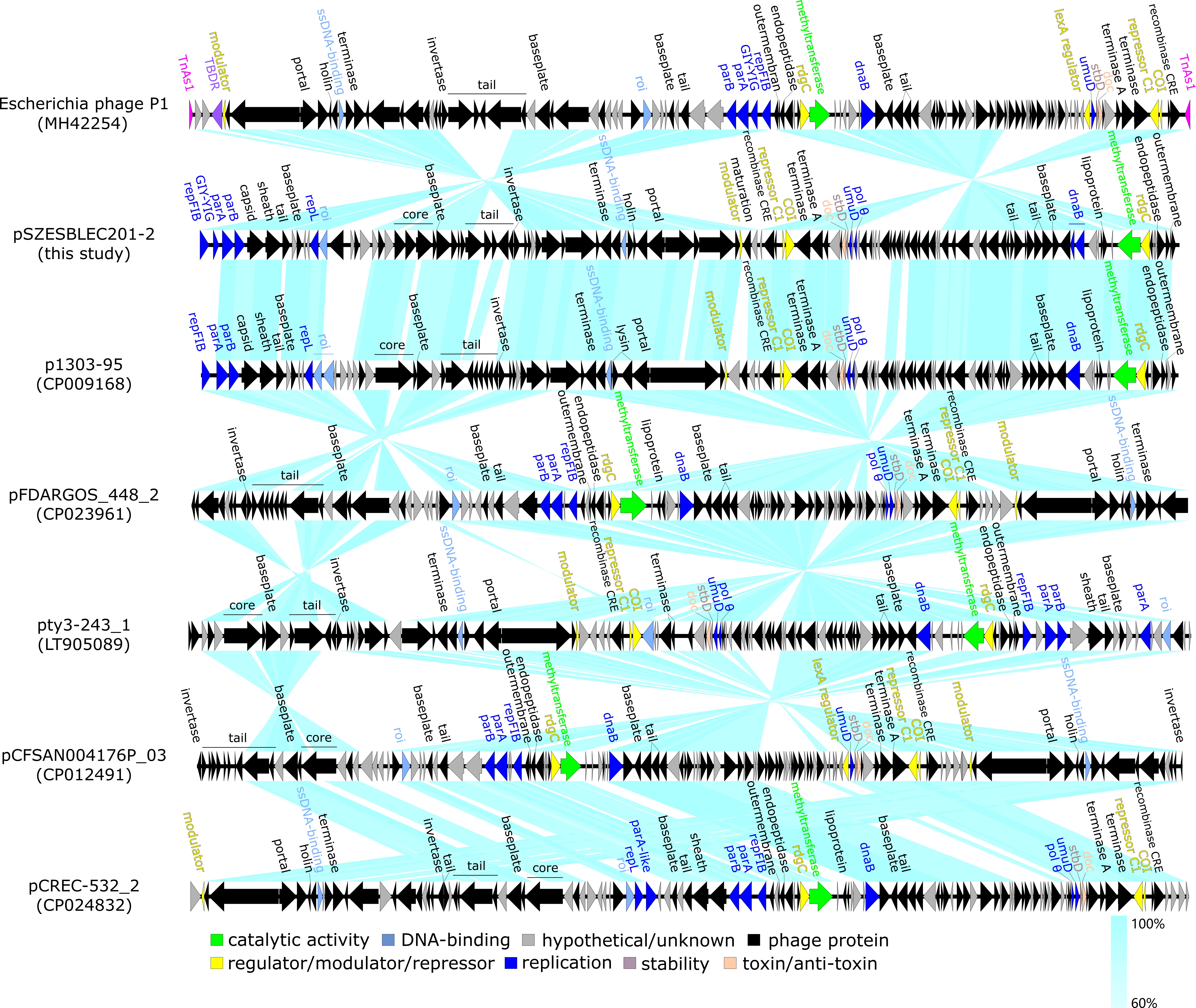

Supplement: Supplementary file 4 [file Image_4.JPEG]

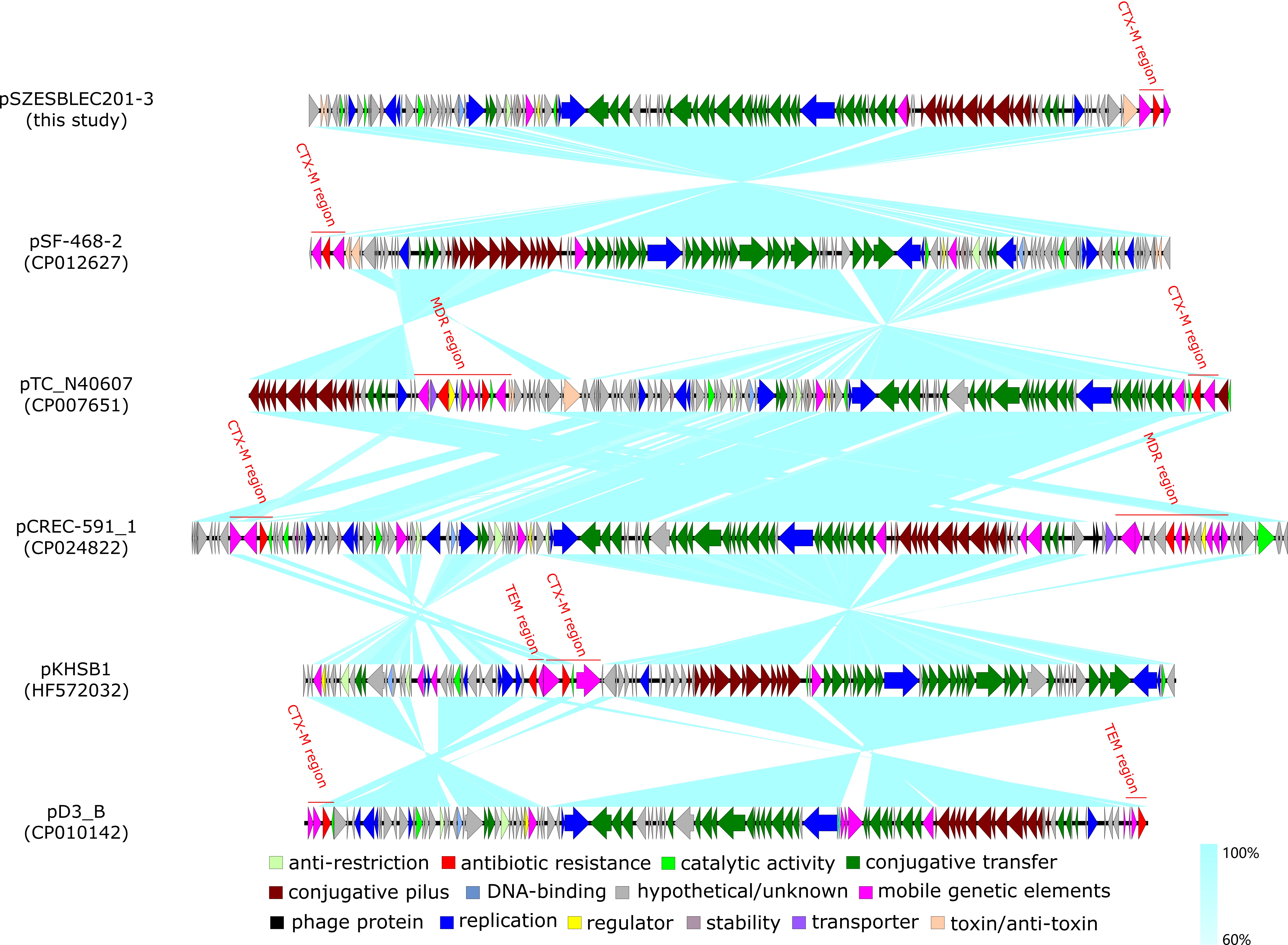

Supplement: Supplementary file 5 [file Image_5.JPEG]

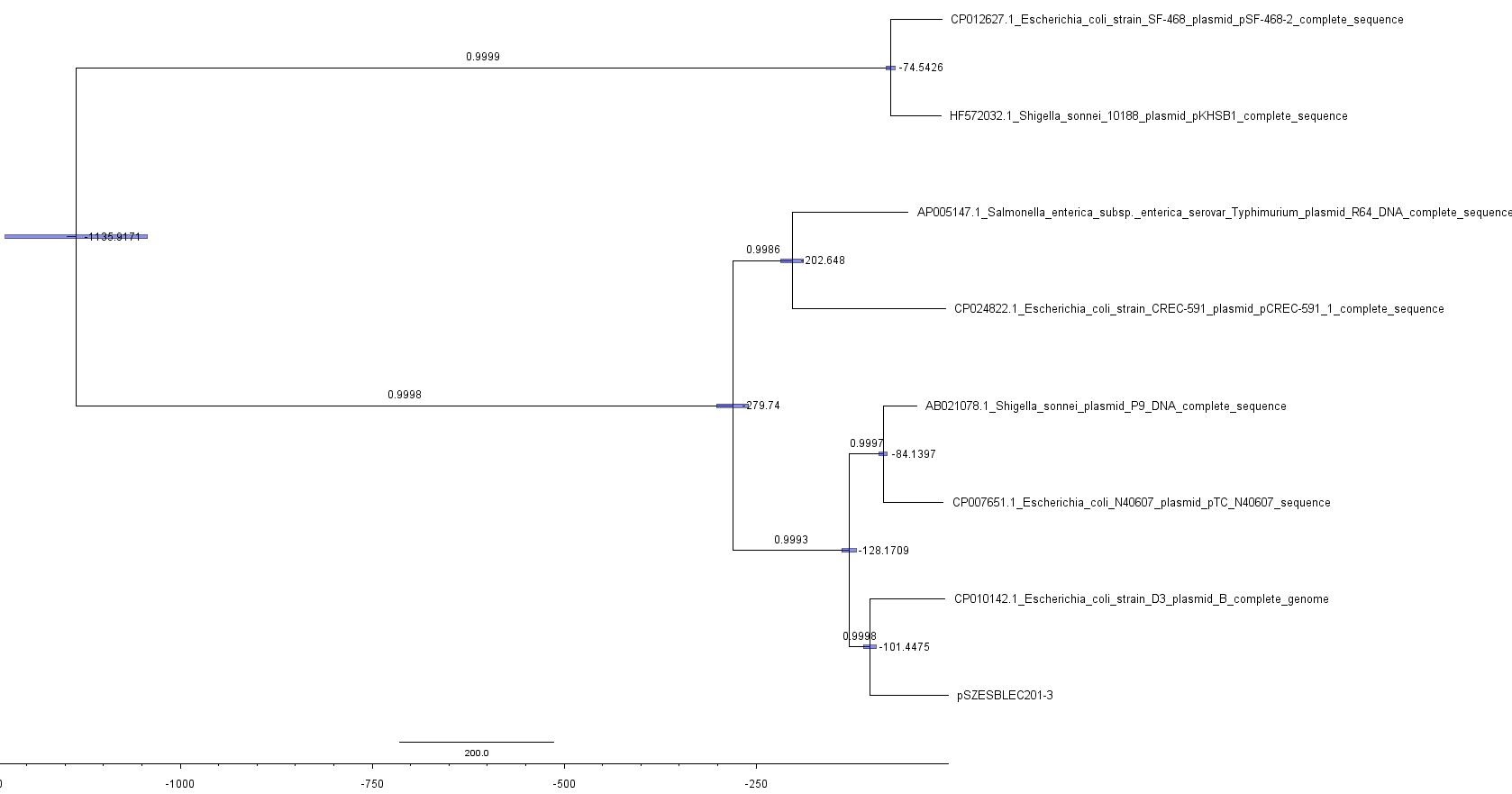

Supplement: Supplementary file 6 [file Image_6.JPEG]
