# Supplementary material for: Genomic Characterization of a Uropathogenic Escherichia coli ST405 Isolate Harboring blaCTX-M-15-Encoding IncFIA-FIB Plasmid, blaCTX-M-24-Encoding IncI1 Plasmid, and Phage-Like Plasmid
Source: Front Microbiol. 2022 Apr 11;13:845045. doi: 10.3389/fmicb.2022.845045 (PMC9037040; doi:10.3389/fmicb.2022.845045)
Supplement: Supplementary file 7 [file Data_Sheet_1.zip › Supplementary figure captions.docx]

**Supplementary Figure S1**. S1-PFGE of plasmid pattern and Southern blot for strain SZESBLEC201. Black arrow indicates plasmid pSZESBLEC201-1; red arrow indicates Southern blot hybridization with probe specific to *bla*_CTX-M_ Group 1.

**Supplementary Figure S2.** S1-PFGE of plasmid pattern and Southern blot for strain SZESBLEC201. Black arrow indicates plasmid pSZESBLEC201-1 and pSZESBLEC201-2; red arrow indicates Southern blot hybridization with probe specific to RepFIB replicon.

**Supplementary Figure S3.** A comparative schematic representation of entire plasmid sequences of pSZESBLEC201-1 (111,621 bp) with plasmid pECO-824 (121,385 bp), uk_P46212 (143,748 bp), AR_0137 (169,449 bp), pEC958 (172,588 bp), and AR_0014 (135,602 bp). Open reading frames are indicated with arrows. Homologous regions are linked with turquoise coloring, and degrees of homology are denoted with color depth. Genes with similar annotated functions are indicated with same color.

**Supplementary Figure S4.** A comparative schematic representation of entire plasmid sequences of pSZESBLEC201-2 (95,138 bp) with plasmid p1303-95 (94,959 bp), pFDARGOS_448_2 (96,807 bp), pty3_243_1 (97,394 bp), pCFSAN004176P_03 (95,721 bp), and pCREC-532_2 (96,987 bp), with Escherichia phage P1 (97, 213 bp) as reference genome. Open reading frames are indicated with arrows. Homologous regions are linked with turquoise coloring, and degrees of homology are denoted with color depth. Genes with similar annotated functions are indicated with same color.

**Supplementary Figure S5.** A comparative schematic representation of entire plasmid sequence of pSZESBLEC201-3 (92,865 bp) with plasmid pSF-468-2 (92,766 bp), pTC_N40607 (105,866 bp), pCREC-591_1 (118,156 bp), pKHSB1 (94,089 bp), and pD3_B (90, 974 bp). Open reading frames are indicated with arrows. Homologous regions are linked with turquoise coloring, and degrees of homology are denoted with color depth. Genes with similar annotated functions are indicated with same color.

**Supplementary Figure S6.** Time-measured phylogeny Tree of plasmid sequences of pSZESBLEC201-3 and its closest related plasmids. Well-documented IncI1 prototype plasmids R64 and ColIb-P9 are included as reference. Scale bar indicates age in years. Node labels indicate node ages in years. Branch labels indicate the posterior for each node.
