# Supplementary material for: Genomic Characterization of a Uropathogenic Escherichia coli ST405 Isolate Harboring blaCTX-M-15-Encoding IncFIA-FIB Plasmid, blaCTX-M-24-Encoding IncI1 Plasmid, and Phage-Like Plasmid
Source: Front Microbiol. 2022 Apr 11;13:845045. doi: 10.3389/fmicb.2022.845045 (PMC9037040; doi:10.3389/fmicb.2022.845045)
Supplement: Supplementary file 7 [file Data_Sheet_1.zip › Supplementary table 1-3.docx]

**Supplementary Table S1.** List of PCR primers used

| **Primers** | **Oligonucleotide sequences (5’-3’)** | **Application** | **References** |
| --- | --- | --- | --- |
| M13U | GGTTAAAAAATCACTGCGTC | *bla*_CTX-M_ Group 1 specific probe synthesis | (Guessennd et al., 2008) |
| M13L | TTGGTGACGATTTTAGCCGC |  |  |
| M9U | ATGGTGACAAAGAGAGTGCA | *bla*_CTX-M_ Group 9 specific probe synthesis |  |
| M9L | CCCTTCGGCGATGATTCTC |  |  |
| FIB-F | GGAGTTCTGACACACGATTTTCTG | PBRT | (Carattoli et al., 2014) |
| FIB-R | CTCCCGTCGCTTCAGGGCATT |  |  |

**REFERENCES**

Carattoli, A., Zankari, E., Garcia-Fernandez, A., Voldby Larsen, M., Lund, O., Villa, L., Moller Aarestrup, F., and Hasman, H. (2014). In silico detection and typing of plasmids using PlasmidFinder and plasmid multilocus sequence typing. *Antimicrob Agents Chemother* 58**,** 3895-3903.

Guessennd, N., Bremont, S., Gbonon, V., Kacou-Ndouba, A., Ekaza, E., Lambert, T., Dosso, M., and Courvalin, P. (2008). [Qnr-type quinolone resistance in extended-spectrum beta-lactamase producing enterobacteria in Abidjan, Ivory Coast]. *Pathol Biol (Paris)* 56**,** 439-446.

**Supplementary Table S2**. Broth microdilution method determined antibiotic resistance profiles (MIC, μg/mL) of SZESBL201 strain, *E.coli* J53 strain and the*E. coli* J53 transconjugant harboring plasmid pSZESBLEC201-3.

| Antibiotics^a^ | SZESBL201  (Donor) | *E. coli* J53  (Recipient) | TcJ53-CTX-M  (Transconjugant) |
| --- | --- | --- | --- |
| AM | >16 (R) | <0.5 (S) | >16 (R) |
| AMC | 8/4 (S) | <1/0.5(S) | <1/0.5(S) |
| AN | 16 (S) | 2(S) | 2(S) |
| ATM | >16 (R) | 0.5(S) | >16 (R) |
| C | ≤4 (S) | 2(S) | 2(S) |
| CAZ | >16 (R) | <1(S) | >16 (R) |
| CIP | >1 (R) | <0.25(S) | <0.25(S) |
| CTX | >32 (R) | <0.5(S) | >32 (R) |
| CZ | >16 (R) | 8(S) | >16 (R) |
| FEP | >16 (R) | <0.5(S) | >16 (R) |
| GM | >8 (R) | <1(S) | <1(S) |
| IPM | ≤1 (S) | <0.5(S) | <0.5(S) |
| LVX | >8 (R) | <0.5(S) | <0.5(S) |
| MEM | ≤1 (S) | <0.5(S) | <0.5(S) |
| PIP | >64 (R) | 4(S) | >64 (R) |
| SAM | 8/4 (S) | <2/0.5(S) | <2/0.5(S) |
| SCF^b^ | 27 (S) | 27 (S) | 27 (S) |
| SXT | >2/38 (R) | <0.5/10(S) | >2/38 (S) |
| TE | >8 (R) | <1(S) | <1(S) |
| TZP | ≤4/4 (S) | <4/0.5(S) | <4/0.5(S) |

^a^AM, Ampicillin; AMC, Amoxicillin/clavulanic acid; AN, Amikacin; ATM, Aztreonam; C, Chloramphenicol; CAZ, Ceftazidime; CIP, Ciprofloxacin; CTX, Cefotaxime; CZ, Cefazolin; FEP, Cefepime; GM, Gentamicin; IPM, Imipenem; LVX, Levofloxacin; MEM, Meropenem; PIP, Piperacillin; SAM, Ampicillin/sulbactam; SCF, Cefoperazone/sulbactam; SXT,Trimethoprim/sulfamethoxazole; TE, tetracycline; TZP, Piperacillin/tazobactam.

^b^Determined by Kirby-Bauer Disk Susceptibility Test and number represented zone diameter (MM). Breakpoints were interpreted according to the manufacture’s recommendations (Bio-Rad, Marnes-la-Coquette, France).

R, resistant; S, susceptible.

**Supplementary Table S3**. General genomic features of the SZESBLEC201 strain

| Complete genome | Length (bp) | No. of coding sequences | GC% | MLST, RST, or pMLST | Plasmid Inc type | Antimicrobial resistance genes | Data Accessibility |
| --- | --- | --- | --- | --- | --- | --- | --- |
| Chromosome | 5,020,403 | 5,114 | 50.79 | ST405 | NA | *mdf*A, *gyr*A(p.S83L and p.D87N), *par*C (p.S80I), *par*E (S458A) | CP090074 |
| pSZESBLEC201-1 | 111,621 | 168 | 51.25 | F-:A1:B1 | IncFIA-FIB | *bla*_CTX-M-15_, *sul*1,*aac*(3’)-IIa, *aad*A5, *dfr*A17, *tet*(B) | CP090075 |
| pSZESBLEC201-2 | 95,138 | 122 | 48.06 | NA | P0111-like | Not detected | CP090076 |
| pSZESBLEC201-3 | 92,865 | 128 | 49.86 | 166 | IncI1 | *bla*_CTX-M-24_ | CP090077 |
